# Frequency and clinicopathological correlation of gastrointestinal polyps: A six-year single center experience

**DOI:** 10.1515/med-2024-1022

**Published:** 2024-09-02

**Authors:** Goran Mohammed Raouf Abdulqader

**Affiliations:** Department of Basic Medical Sciences, College of Medicine, University of Sulaimani, 0046 Sulaimaniyah, Iraq

**Keywords:** adenoma, colorectal carcinoma, colonoscopy, polyps, retrospective study

## Abstract

**Background:**

Most gastrointestinal polyps are asymptomatic; therefore, assessing symptoms associated with cancer and precancerous polyps is essential.

**Objectives:**

The aim of this study was to study the histopathology, number, distribution, and degree of polyps’ dysplasia in terms of age, gender, and clinical presentation.

**Methods:**

This study was performed on patients who underwent endoscopy from July 2015 to August 2021 in Sulaimaniyah, Iraq. Surgical pathology records of patients were analyzed for age, sex, nature of the polyps, number, site, histopathology, degree of dysplasia, resection margins and patients’ presented clinical data.

**Results:**

The mean patients’ age was 51.4 ± 17.1 years, and most were males (51.9%). The most common indications for endoscopy were screening (28.62%), and the least common was weight loss (4.46%). Neoplastic polyps were common among patients with hematemesis (75%), while non-neoplastic were common among those with dyspepsia (60%). Most polyps were solitary in upper (80.8%) and lower gastrointestinal tract (GIT). Most polyps in the upper GIT were non-neoplastic (87.3%), while most lower proximal/distal GIT polyps were neoplastic. Most neoplastic polyps showed low-grade dysplasia (94.4%), and most high-grade dysplasia was a villous type (24.1%).

**Conclusions:**

Initiation of the screening program is highly recommended as a facilitating method for the early detection of multiple and high-grade gastrointestinal polyps. Thus, screening programs can reduce the rate of mortality of carcinoma in this locality.

## 1 Introduction

Polyp originated from the Greek word polypous, which means morbid lump [1], and esophagus-gastrointestinal (GI) polyps are one of the most common pathologies affecting gastrointestinal tract (GIT). Most gastric polyps do not have a counterpart in the large bowel. Gastric polyps include hyperplastic, adenoma, pyloric gland adenoma, oxyntic gland adenoma, fundic gland polyps, and inflammatory fibroid polyp [2].

Duodenal polyps’ prevalence is estimated to be about 1% in patients referred for esophagus-gastro-duodenoscopy (EGD), including inflammatory polyps, Brunner’s gland hyperplasia, and those with ectopic gastric mucosa [3]. Polyps of the small bowel are rare compared to those of the colo-rectum, with adenomas being most common and having more preference for the distal duodenum, ampullary, and periampullary region [4].

Colorectal cancer (CRC) is one of the most common cancers that annually affects over 1.23 million people worldwide, which accounts for 10% of all malignancies [5]. Most CRCs are believed to arise from adenomas, benign precursor lesions that can develop malignant transformation through genetic and epigenetic mutations, the adenoma-carcinoma sequence [6]. The progression from detectable adenomas to cancer requires at least 10–15 years, leaving a reasonable period for endoscopic resection at the premalignant stage [7]. The time from initial invasive cancer to clinically overt disease may take several years. Thus, the long premalignant and preclinical course makes CRC a candidate for screening [8].

Histologically, intestinal polyps can be classified into neoplastic and non-neoplastic polyps, with the most common neoplastic types being colonic adenomas. In contrast, the non-neoplastic polyps can be hamartomatous, hyperplastic, or inflammatory polyps or subtypes [9]. Among GIT malignancies, CRC has the most excellent chance of curability as far as it is discovered at an early stage, either as a premalignant lesion or before lymph node metastasis [6]. In European national screening programs, around 17% of cancers were pT1 CRCs, and the risk of having advanced neoplasia following polypectomy was found to be 0.6% [10].

Approximately 30% of Western people have colon polyps, while 10–15% is reported for Asians and Africans, with more frequency in males and aged people [11]. Evaluating the site and histological types in colorectal polyps and cancer is essential since these are associated with the efficacy of colorectal screening strategies [12]. The colorectal polyp management is guided by histological features of the polyp (presence/absence of dysplasia) in a way that adenomatous polyp requires follow-up assessment. In contrast, non-adenomatous polyps usually do not [2]. Colonoscopy is considered the gold standard screening tool for early detection of colon cancer by facilitating the removal of precancerous adenomas [13]. The incidence and mortality of CRC are decreasing due to effective screening programs [7].

Recently, many countries, in addition to adopting national CRC screening programs, have been pursuing strategies to increase the participation of suitable individuals in these programs. A one-time colonoscopy screening at the age of 55 could achieve a 30–50% reduction in mortality from CRC [14]. The costs of performing colonoscopies clinically and financially are high; it is therefore essential to assess which symptoms are associated with cancer and precancerous polyps. Bowel symptoms, including alteration in bowel habits, rectal bleeding, abdominal pain, and weight loss, are often considered an indication to perform a colonoscopy to identify or rule out CRC or precancerous polyps [15].

Thus, to lower the incidence and mortality of CRC in the Kurdistan region of Iraq, the current study was conducted to determine the age- and sex-related frequency of gastrointestinal polyps, the most common clinical presentation, and its relation to the histological types. Further, the degree of dysplasia and the excisional margins of the polyps were evaluated.

## 2 Patients and methods

### 2.1 Study design and setting

The current study was performed retrospectively, and the clinical data (reason for visiting or referral) to Kurdistan Center of Gastroenterology and Hepatology (KCGH) of only 1,055 out of the 1,764 patients were available in the patient’s records. Thus, we used the records of 1,764 patients who underwent endoscopy procedures, including EGD and colonoscopy in KCGH, Sulaimaniyah, Iraq, from 1st July 2015 to 31st August 2021 (over 6 years).

### 2.2 Study population

The study population was patients who visited or were referred to the KCGH with upper GIT symptoms like epigastric pain, dyspepsia, or lower GIT symptoms such as diarrhea, abdominal pain, bleeding per rectum, and those who complained of unexplained anemia, weight loss, or those who referred for surveillance/screening colonoscopy.

### 2.3 Data collection

Official permission was taken from the authorities to collect the patients’ data from the hospital database, such as age, sex, presented clinical data, nature of the polyps (neoplastic vs non-neoplastic), number (solitary or multiple), site, histopathological type, degree of dysplasia, and resection margins was saved and sorted in an access file.

### 2.4 Anatomical distribution of polyps

The anatomical location of the polyp was divided into upper GIT (esophagus, stomach, duodenum till ileum), proximal colon (cecum, ascending, hepatic flexure, and transverse colon), and distal colon (splenic flexure, descending, sigmoid colon, and rectum).

### 2.5 Histopathological analysis

The endoscopically resected polyps were received in 10% buffered neutral formalin, processed by Leica auto processor TP1020 for 24 h, cut into 5.0 µm sections using Accu-Cut SRM 200 rotary microtome and stained manually with hematoxylin and eosin (H&E). Lastly, the histopathological findings were reported by a GIT pathologist. World Health Organization criteria were applied to classify the polyps into non-adenomatous (hyperplastic and inflammatory) and adenomatous (tubular, tubulovillous, villous, and sessile serrated lesion) according to their architecture [16].

### 2.6 Inclusion criteria

Patients with a history of upper/lower GIT malignancy, including familial adenomatous polyposis, previous polyps, inflammatory bowel disease, positive fecal occult blood, history of upper GIT bleeding, other malignancies outside the GIT, and history of chronic liver disease.

### 2.7 Exclusion criteria

Concerning patients presented with multiple polyps, the dysplastic polyps with the highest grade were considered in the analysis while lower grades or non-neoplastic ones were not considered since it was impossible statistically to consider every single polyp.

### 2.8 Statistical analysis

Statistical Package for Social Science (SPSS, Armonk, NY: IBM Corp, USA, version 25) was used for data interpretation. Descriptive statistics were used to measure the data using frequency, proportion, mean, and standard deviation. The chi-square test was applied to detect the difference between categorical data, while the Fisher exact test was used when >20% of cells have expected frequencies of <5.0. A *p*-value of <0.05 was considered statistically significant.


**Ethical approval:** The Scientific and Ethical Committees approved the proposal for the present study at the College of Medicine, University of Sulaimani, Sulaimaniyah, Iraq. Also, official permission was taken from the KCHG authorities.

## 3 Results

The patients’ ages ranged from 1.0 to 97 years old, with a mean range of 51.4 ± 17.1 years and a median (interquartile range) of 52 (23). Most patients (76.4%) were in the age group of 31–70 (Table 1) and were males (51.9%). The types of polyps involved in this study were listed in the supplementary file. The most common indications for performing EGD and colonoscopy in the patients were screening (28.62%), bleeding per rectum (25.97%), chronic abdominal pain (12.6%), constipation (10.14%), chronic non-bloody diarrhea (3.22%), and weight loss (4.46%). However, none of these symptoms was significant compared to the age groups (Table 2).

**Table 1:** The age distribution of the studied patients

Age (years)	Number	%
1–10	45	2.6
11–20	26	1.5
21–30	134	7.6
31–40	232	13.2
41–50	364	20.6
51–60	374	21.2
61–70	378	21.4
71–80	167	9.5
81–90	39	2.2
91–100	5.0	0.3
Total	1,764	100

**Table 2:** The clinical presentations among the studied patients about the age group

Variable	Age (years)		Total		*p*-value
	<50	≥50	No.	%	
**(A) Indication for colonoscopy**					
Chronic abdominal pain	49	84	133	12.6	0.06
Bleeding per rectum	118	156	274	25.97	
Chronic diarrhea (non-bloody)	14	20	34	3.22	
Bloating	9.0	8.0	17	1.61	
Constipation	49	58	107	10.14	
Melena	6.0	4.0	10	0.95	
Alteration of bowel habit	13	11	24	2.27	
Anemia	12	18	30	2.84	
Weight loss	20	25	45	4.26	
Surveillance/follow-up	125	177	302	28.62	
**(B) Indication for EGD**					
Dysphagia	1.0	2.0	3.0	0.28	0.72
Vomiting	0.0	5.0	5.0	0.47	
Hematemesis	2.0	6.0	8.0	0.76	
Dyspepsia	8.0	7.0	15	1.42	
Epigastric pain	9.0	24	33	3.13	
Anorexia	2.0	5.0	7.0	0.66	
Anemia	1.0	4.0	5.0	0.47	
Weight loss	0.0	3.0	3.0	0.28	
Total	438	617	1055	100	

EGD: esophagus-gastro-duodenoscopy.

**Figure 1:**
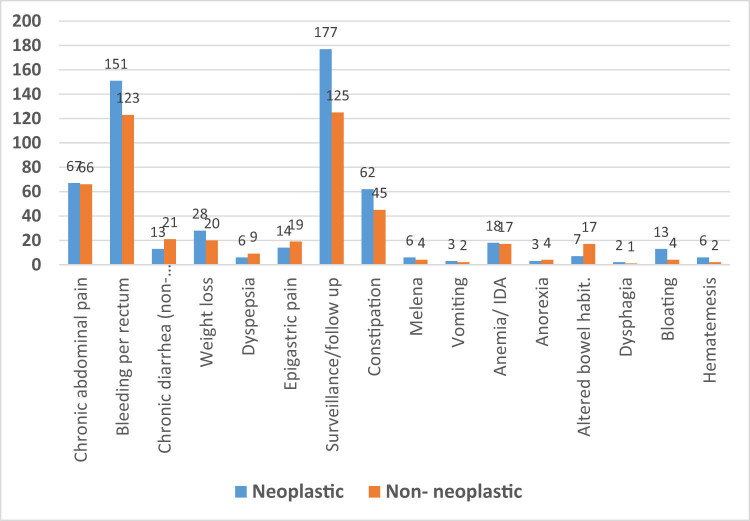
Correlation of clinical presentations of the studied patients with their types of polyps (neoplastic vs non-neoplastic).

Moreover, there was no significant correlation between patients’ clinical presentations about the types of polyps (*p* = 0.10). However, neoplastic polyps were common in patients with hematemesis (75%), bloating (75.5%), dysphagia (66.7%), routine screening (58.6%), weight loss (58.3%), and constipation (57.9%). While non-neoplastic polyps were common in patients with dyspepsia (60%), epigastric pain (57.6%), chronic diarrhea (61.8%), and altered bowel habits (70.8%), on the other hand, some categories that were expected to be significantly higher in neoplastic polyps were surprisingly having slightly higher percent compared to non-neoplastic polyps like bleeding per rectum (55.1% in neoplastic vs 44.9% in non-neoplastic) and anemia (51.4% in neoplastic vs 48.6% in non-neoplastic) (Figure 1).

**Figure 2:**
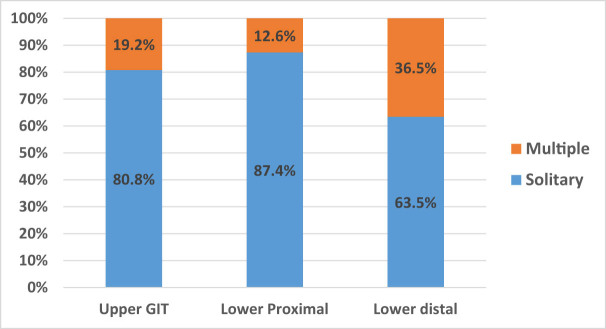
Shows the polyp’s number about its location in the GIT (*p* < 0.001).

**Figure 3:**
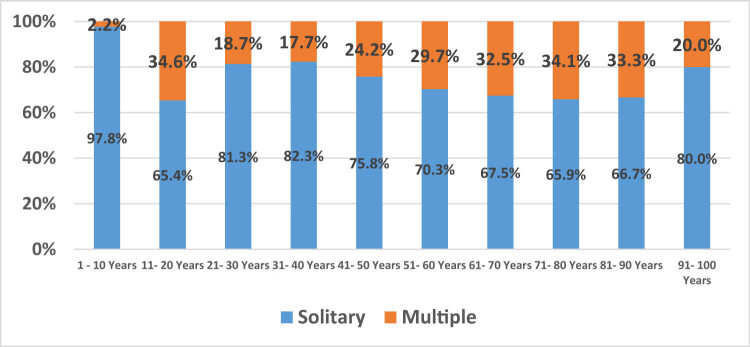
Illustrates the percentage of solitary and multiple polyps in different age groups.

**Figure 4:**
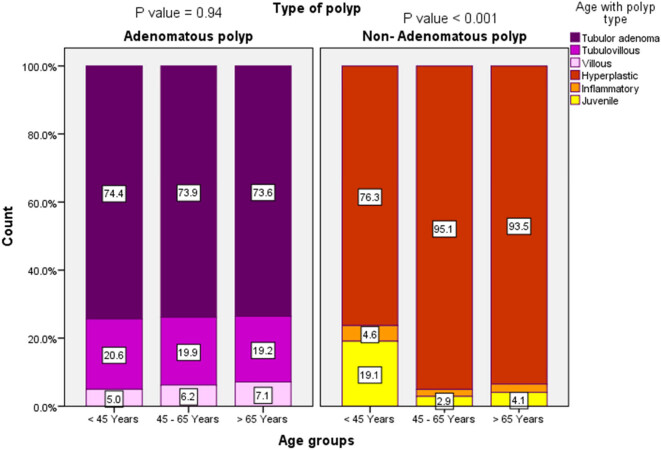
The most frequent polyps in comparison to various age groups.

Furthermore, there was a significant difference between the site and the number of the polyps, with the majority of them being solitary in upper (80.8%) and lower GIT (87.4% in lower proximal and 63.5% in lower distal) (*p* < 0.001) (Figure 2). The multiple polyps in the various age groups were 2.2–34.6%, with the highest numerous polyps being in the 11–20 years (34.6%) (Figure 3). There was no association between age and subtype distribution among adenomatous polyps (*p* = 0.94). Meanwhile, a significant association between non-adenomatous subtypes and different age groups was found (*p* = 0.001) (Figure 4).

**Figure 5:**
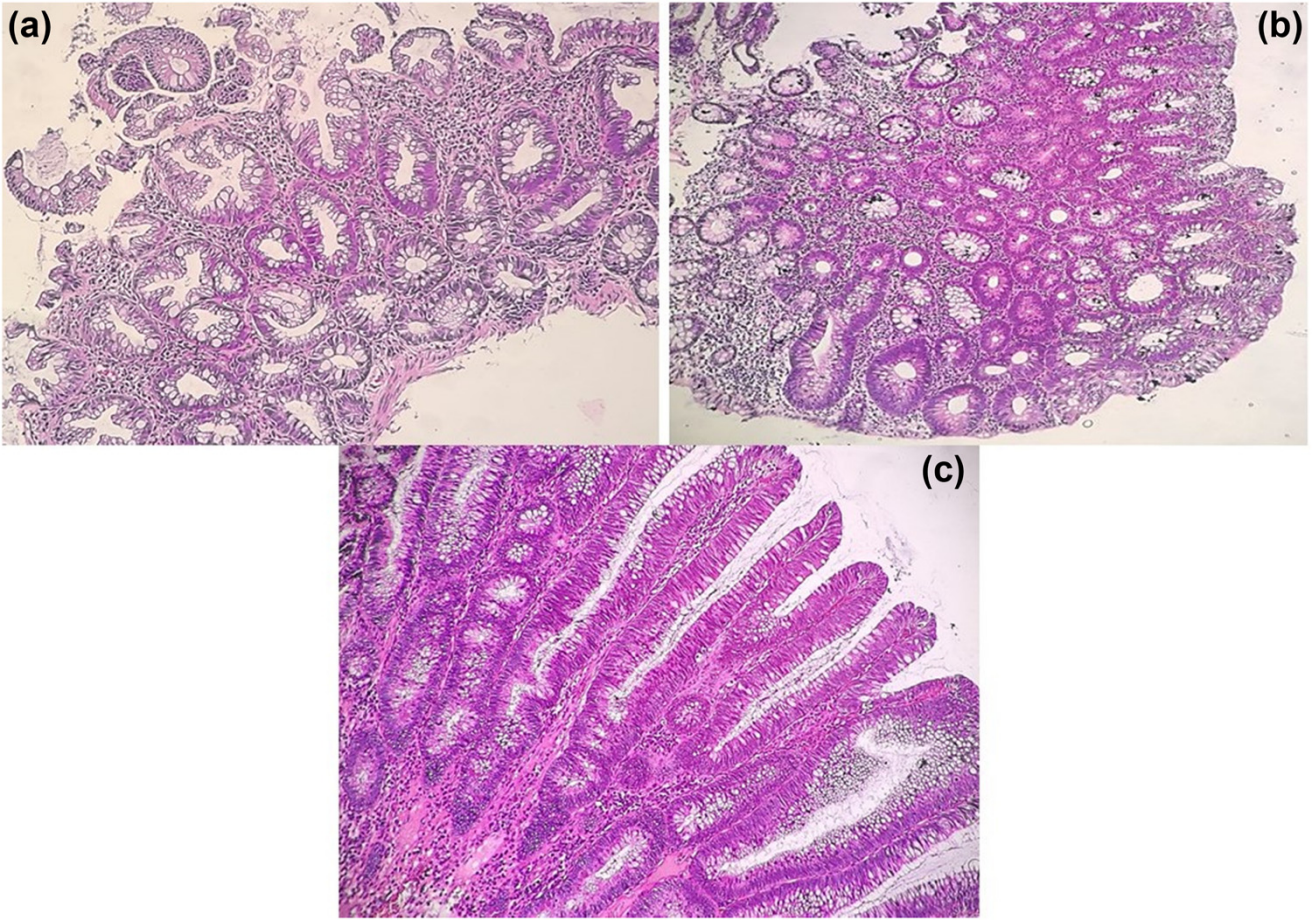
Colonic hyperplastic polyp, goblet cell-rich type (a), colonic tubular adenoma, low-grade dysplasia (b), and colonic villous adenoma with low-grade dysplasia (c). H&E-100 HPF.

Additionally, there was no significant finding between the degree of dysplasia and the polyp’s number. However, most polyps with low- (64%) and high-grade (64.58%) dysplasia were solitary (*p* = 0.97) (Table 3). Most polyps in the upper GIT were non-neoplastic (87.3%), while most of the lower proximal (65.6%) and distal GIT polyps were neoplastic (51%) (*p* < 0.001) with tubular adenoma been the frequent subtype (Table 4). Although most of the neoplastic polyps (tubular, tubulovillous, and villous) showed low-grade dysplasia (94.4%), most high-grade dysplasia was a villous type (24.1%) (*p* = 0.001) (Table 5 and Figure 5). Concerning the resection margin of the neoplastic polyps, the majority (67.5%) were wholly excised, 9.5% were incompletely excised, and 21.6% were fragmented polyp specimens (Table 6).

**Table 3:** The relation between the degree of dysplasia among the neoplastic polyps and the number of polyps

Degrees of dysplasia	Polyp number (%)			*p*-value
	Solitary	Multiple	Total	
Low grade	522 (64%)	293 (36%)	815 (100%)	0.97
High grade	31 (64.58%)	17 (35.42%)	48 (100%)	
Total	553 (64.08%)	310 (35.92%)	863 (100%)	

**Table 4:** The number of neoplastic and non-neoplastic polyps in different GIT sites

Site	Neoplastic	Non-neoplastic	Total	p-value
	Number (%)			
Upper GIT	33 (12.7)	227 (87.3)	260 (100)	<0.001**
Lower proximal	358 (65.6)	188 (34.4)	546 (100)	
Lower distal	489 (51.0)	469 (49.0)	958 (100)	
Total	880 (49.9)	884 (50.1)	1,764 (100)	

**Highly significant difference.

**Table 5:** The degree of dysplasia in different types of neoplastic polyps

Types of polyps	Low grade		High grade		Total	*p*-value
	Frequency	%	Frequency	%		
Tubular adenoma	627	98.6	9.0	1.4	636	<0.001**
Tubulovilous adenoma	144	85.2	25	14.8	169	<0.001**
Villous	44	75.9	14	24.1	58	<0.001**
Total	815	94.4	48	5.6	863	

**Highly significant difference.

**Table 6:** Excisional margins of the neoplastic polyps

Excisional margin	Frequency	%
Completely excised	594	67.5
Incompletely excised	84	9.5
Very close	12	1.4
Fragmented (cannot be assessed)	190	21.6
Total	880	100

**Figure 6:**
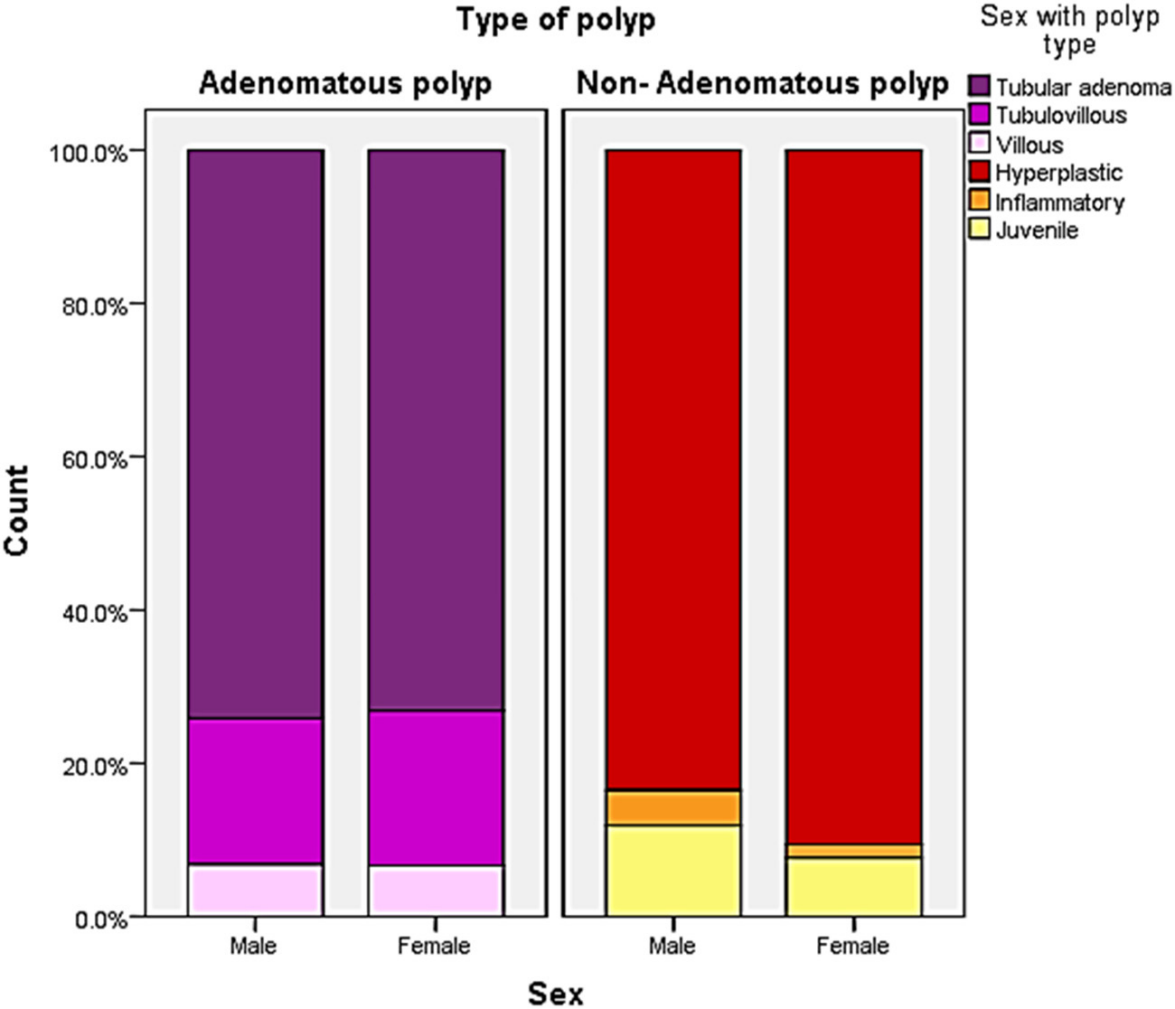
The distribution of the type of polyps about the gender in the studied cases.

**Figure 7:**
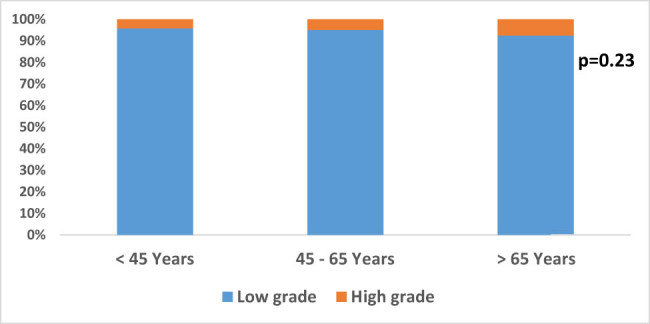
The degree of dysplasia in different age groups.

**Figure 8:**
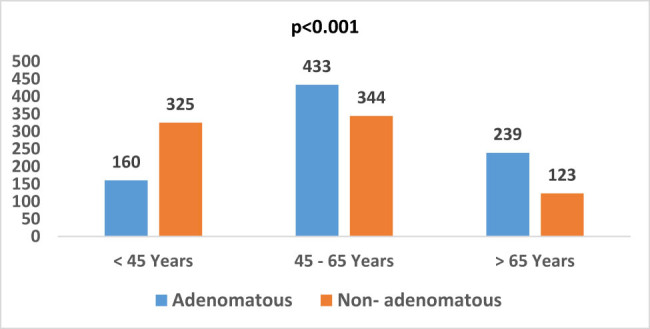
Distribution of types of polyps in different age groups.

A significant gender difference was observed among patients with non-adenomatous polyps, such that the non-adenomatous polyps were more frequent among females (*p* = 0.01). In contrast, no significant difference was observed among patients with adenomatous polyps (*p* = 0.92) (Figure 6). There was no correlation between the degree of dysplasia and different age groups (*p* = 0.23) (Figure 7). Moreover, most of the adenomatous polyps were in the age group of 45–65 years, with a significantly lower trend in the two extremes (<45 and >65 years) (*p* = 0.001); however, in the non-adenomatous polyps, the number of patients in age group >65 years was significantly lower compared to other two groups (Figure 8).

## 4 Discussion

CRC is the third most common cancer worldwide, and 80% of CRCs arise from preexisting adenomas [17]. Colorectal polyps have had an increasing detection rate in recent years owing to the fact that they are a recognized premalignant lesion of CRC [18]. Colonoscopy is universally used for both the diagnosis and treatment of colon pathologies, and it is considered safe, accurate, and well-tolerated if properly performed [19].

In the present study, the mean age of patients was 51.4 ± 17.1 years, slightly younger than a study reported by Sohrabi et al. (56.45 ± 9.59 years) [20]. Our reported mean age was potentially lower than that stated by Nam et al. (64.1 ± 12.5 years) [11] but close to that found by Almadi et al. (50.5 ± 15.9 years) [21]. The Westernization of our community’s lifestyle can explain this. The incidence of polyps increased with age; the three age groups from 41 to 70 years had approximately similar percentages (20.6, 21.2, and 21.4%, respectively). These data are lower than the age groups presented for polypectomy by Sohrabi et al. (26.8, 29.7, and 31.4%, respectively) [20].

Additionally, there was no gender difference among patients with adenomatous polyps, although most were males (472 patients, 54.6%), similar to earlier studies [20,22]. Also, men had more adenomatous polyps than women, which agrees with that of McCashland et al. [23], who found that men had a greater risk of polyps and tumors.

Concerning indication for colonoscopy, 28.6% presented with bleeding per rectum, 13.9% with chronic abdominal pain, and 11.2% with constipation, which is near to that reported by Almadi et al. (25.1, 20.3, and 10.8%, respectively) [21]. Also, 31.5% of the patients had undergone screening colonoscopy, contrary to 7.7% reported by Almadi et al. [21]. Our study’s high percentage of screening was because we included many presentations in the screening group. Also, we found that most non-adenomatous polyps were hyperplastic and found in patients aged >45 years (*p* = 0.001). At the same time, there was no significant difference regarding adenomatous polyps with age distribution (*p* = 0.94). This contradicts Cekodhima et al., in which tubular adenoma drops and villous adenoma increases with advanced age [24].

In the current study, most patients had multiple polyps in the distal colon (36.5%), similar to a survey by Mehdi, 2021 (32.2%) [25]. All age groups had numerous multiple polyps, except age group 1.0–10 years, and only 2.2% of them had multiple polyps. These are significantly higher compared to Mehdi’s study in 2021 [25]. This can be explained by the fact that we used a large sample size (1,764 cases). The average of multiple polyps in the proximal and distal colon was 24.5%, which is in agreement with Al-jameel et al., who reported that 25.9% of colonic polyps were multiple [26].

Both groups of patients with solitary and multiple polyps showed almost a similar degree of dysplasia. Hence, there was no significant difference between solitary and various polyps concerning the increased risk of high-grade dysplasia development. Furthermore, 2/3 of the cases had solitary polyps, similar to Lowenfels et al. [27] but higher than Silva et al., who reported that almost 50% of cases had solitary polyps [28].

In the current study, 63.6% of patients had polyps at the distal colon, which is slightly lower than Long et al., who reported 66.4% [18] and Bafandeh et al., who reported 72% [29] and even lower than Al-jameel et al. who reported 70% [26]. On the other hand, our finding was higher than Almadi et al.‘s, who reported 55.5% [21], and higher than Nam et al.‘s (53.4%) [11]. These discrepancies between our findings may be explained by factors like diet and lifestyle habits, genetic differences, and improved colonoscopy techniques [30].

Regarding the fragmented polyp specimen (21.6%), these findings indicate a poor endoscopic resection technique, which can be explained by the fact that a significant proportion of endoscopic procedures were performed by trainee endoscopists who had a low level of experience.

In this study, the tubular adenoma subtype accounts for 72.2% of all neoplastic polyps (636/880), which is lower than that reported by Cekodhima et al. (89.2%) [24]. This may be due to the small sample size in the mentioned study (346 polyps) compared to this study (880 polyps). Around 67.5% of the neoplastic polyps were excised entirely, 9.5% were incompletely resected, and fragmented polyps account for 21.6%. This was mainly due to differences in endoscopic techniques applied for polyp resection, endoscopist experience, and inappropriate handling of specimens during polyp resection [22]. Lastly, the significance of the current study is the large sample size regarding the number of different variables measured and compared to each other, which were not done previously in our locality.

## 5 Conclusions

The most common indication for polypectomy was routine screening. Most polyps were solitary, and multiple polyps were mainly in the distal colon. The upper GI polyps were almost non-neoplastic, while the lower GI polyps were mostly neoplastic. Therefore, routine colonoscopy is highly recommended as a preventive and effective measure to reduce the chance of CRC in our locality. The strength of this study stays in its large sample size; however, including symptomatic patients in a single center is a less representative sample. Future directions for further research in the field include involving more centers and even the private sector in such studies.
